# Incidence and risk factors for HIV-tuberculosis coinfection in the Cologne–Bonn region: a retrospective cohort study

**DOI:** 10.1007/s15010-024-02215-y

**Published:** 2024-03-16

**Authors:** Isabelle Suárez, Dominic Rauschning, Cora Schüller, Anna Hagemeier, Melanie Stecher, Clara Lehmann, Philipp Schommers, Stefan Schlabe, Jörg-Janne Vehreschild, Carolin Koll, Carolynne Schwarze-Zander, Jan-Christian Wasmuth, Angela Klingmüller, Jürgen Kurt Rockstroh, Gerd Fätkenheuer, Christoph Boesecke, Jan Rybniker

**Affiliations:** 1 Department I of Internal Medicine, Medical Faculty and University Hospital of Cologne, University of Cologne, Kerpener Str. 62, 50937 Cologne, Germany; 2 German Center for Infection Research (DZIF), Partner Site Bonn-Cologne, Cologne/Bonn, Germany; 3 Center for Molecular Medicine Cologne, University of Cologne, Cologne, Germany; 4 Department of Medicine I, University Hospital Bonn, Bonn, Germany; 5 Medical Faculty and University Hospital Cologne, Institute of Medical Statistics and Computational Biology, University of Cologne, Cologne, Germany; 6 Medical Department 2 (Hematology/Oncology and Infectious Diseases), Center for Internal Medicine, University Hospital, Goethe University Frankfurt, Frankfurt am Main, Germany; 7 Department IB of Internal Medicine, Bundeswehrzentralkrankenhaus Koblenz, Koblenz, Germany; 8 Gemeinschaftspraxis am Kaiserplatz, Bonn, Germany

**Keywords:** HIV, Tuberculosis, Antiretroviral therapy, TB low-burden country, Germany, Immigrants

## Abstract

**Purpose:**

The risk of developing active tuberculosis (TB) is considerably increased in people living with HIV/AIDS (PLWH). However, incidence of HIV/TB coinfection is difficult to assess as surveillance data are lacking in many countries. Here, we aimed to perform a quantitative analysis of HIV/TB coinfections within the Cologne/Bonn HIV cohort and to determine risk factors for active TB.

**Methods:**

We systematically evaluated data of patients with HIV/TB coinfection between 2006 and 2017. In this retrospective analysis, we compared HIV/TB-coinfected patients with a cohort of HIV-positive patients. The incidence density rate (IDR) was calculated for active TB cases at different time points.

**Results:**

During 2006–2017, 60 out of 4673 PLWH were diagnosed with active TB. Overall IDR was 0.181 cases/100 patient-years and ranged from 0.266 in 2006–2009 to 0.133 in 2014–2017. Patients originating from Sub-Saharan Africa had a significantly (*p* < 0.001) higher IDR (0.694/100 patient-years of observation, 95% CI [0.435–1.050]) in comparison to patients of German origin (0.053/100 patient-years of observation, 95% CI [0.028–0.091]). In terms of TB-free survival, individuals originating from countries with a TB incidence higher than 10/100,000 exhibited a markedly reduced TB-free survival compared to those originating from regions with lower incidence (*p* < 0.001). In 22 patients, TB and HIV infection were diagnosed simultaneously.

**Conclusion:**

Overall, we observed a decline in the incidence density rate (IDR) of HIV/TB coinfections between 2006 and 2017. Patients originating from regions with high incidence bear a higher risk of falling ill with active TB. For PLWH born in Germany, the observed risk of active TB appears to be lower compared to other groups within the cohort. These findings should be considered when developing TB containment and screening strategies for PLWH in low-incidence countries.

**Supplementary Information:**

The online version contains supplementary material available at 10.1007/s15010-024-02215-y.

## Introduction

Tuberculosis (TB) and HIV coinfection (HIV/TB coinfection) represents a severe and life-threatening combination throughout the world [1–4]. Despite large efforts by the World Health Organization (WHO), TB remains the main cause of death for people living with HIV/AIDS (PLWH) [3, 5] with African countries showing the highest burden of HIV-associated TB cases. In 2022, an estimated 10.6 million people worldwide contracted TB, with 6.3% of these cases being HIV positive [3]. During the same year, 167,000 documented deaths were attributed to HIV/TB coinfection, underscoring TB as a prominent opportunistic infection. Globally, PLWH face a 20–30 times higher risk of developing active TB compared to HIV-negative individuals [6–8]. This estimate is often based on studies conducted in high TB burden areas, where factors like TB prevalence, healthcare infrastructure, and public health interventions can significantly influence the risk. It is important to recognize that the risk in Germany may be different, as data estimates regarding HIV/TB coinfection do not exist. Various guidelines recommend testing of all TB patients for HIV [9–11]. In 2022, the global percentage of notified TB cases with a documented HIV test result was 80%. While a high testing coverage of 94% is reported by the WHO for the European region, the most recent data for the European Union published in 2018 show a test rate of 73.4% only, suggesting that the prevalence of HIV among TB patients may be underestimated [2, 12]. National data from Germany concerning the HIV testing coverage were not provided by the WHO or the ECDC.

In Germany, following a minimal rise in 2013, a significant increase of new TB cases was reported in 2015 (7.1 cases per 100,000) that peaked in 2016 (7.2/100,000) [13]. An increasing number of migrants was identified as a main driver of the growing TB incidence in Germany [14]. Although this only affects a short period of time, it is currently unknown whether these incidence trends in TB correlate with the number of HIV/TB coinfections in the country. Despite the crucial importance of assessing longitudinal epidemiological data and risk factors for HIV/TB in every country, precise numbers regarding the incidence of HIV/TB coinfection are not available in Germany, as combined HIV and TB surveillance data sources do not exist [4]. To address this knowledge gap, the present study aimed to analyze the epidemiology of HIV/TB coinfections in a western German metropolitan region using the Cologne/Bonn HIV cohort and focusing on risk factors for active TB and the incidence density rate (IDR) over time.

## Methods

### Study population

The study was performed at the German Center for Infection Research (DZIF) partner sites Cologne/Bonn and involved outpatient departments of two German University Hospitals in Bonn and Cologne. We conducted a retrospective subgroup analysis focusing on HIV/TB coinfection utilizing data from the Cologne/Bonn HIV cohort, which was established in 1996 [15]. The cohort represents an ongoing prospective, observational multicenter study including PLWH treated in the two centers. Patients with HIV/TB coinfection were identified between 2006 and 2017 using ICD-10 codes. Individual patient records were evaluated, and the identified cases were compared to the overall cohort of PLWH treated in Cologne/Bonn during the specified time period. The diagnosis of TB was defined as active TB, i.e., confirmed by cultural and/or molecular biological detection of *Mycobacterium tuberculosis* complex during the observation period. In case of a history of TB documented within the observation period, the diagnosis had to be documented by a physician or health authorities. Diagnosis with active TB prior to the observation period was not taken into consideration for the case group; these patients were registered solely as HIV positive. Data concerning the initial diagnosis of HIV, immune status, status of antiretroviral therapy (ART), place of origin, sex, clinical manifestation of TB, and other clinical characteristics were evaluated for this retrospective analysis. Patients who received antiretroviral therapy (ART) after falling ill with TB were classified as not having received antiretroviral therapy at the time point of TB diagnosis.

### Statistical analysis

The overall observation span covers January 1st 2006 to December 31st 2017. Statistical analyses were performed using GraphPad Prism version 10.0.2 for Windows (GraphPad Software, Boston, Massachusetts USA) and IBM SPSS Statistics for Windows (Version 25.0, NY: IBM Corp). We calculated and reported study characteristics as absolute numbers and percentages, medians plus interquartile range (IQR) or means plus/minus standard deviation (SD), as appropriate. Demanding 95% confidence intervals (95% CI), *p* values < 0.05 were considered statistically significant. Continuous variables such as age at HIV or TB diagnosis were compared by Mann–Whitney *U* test. Categorical variables were analyzed using frequencies and percentages and were compared by *χ*^2^-test for independence. For variables like viral load and CD4 + cell count collected at enrollment, we used the first eligible data within the observation period. When comparing regions of origin, we categorized the cases into different groups: German origin, those originating from Sub-Saharan Africa, and those originating from other countries or countries with an estimated TB incidence of < 10, 10–50, and > 50 per 100,000, according to the 2023 WHO Global TB Report.

If applicable, date of initial TB diagnosis or date of death was determined as end date of the observation. For all other patients, the individual end of observation was determined by the date of the last documented viral load or CD4 cell count findings, serving as a surrogate for continuous clinical care. If this date was after the end of the study (31 Dec 2017), the end of the study corresponded to the end of observation. If the date was before 31 Dec 2017, the end of observation, i.e., lost to follow-up, was set at 3 months after the date of the last documented findings. This decision was based on the expectation that a new presentation of the patient would have occurred by then but not later than 31 Dec 2017 [15, 16]. Kaplan–Meier curves were used to illustrate the TB-free survival from the date of HIV diagnosis. Lost to follow-up was censored. Differences were analyzed by log-rank test with the significance level set to 0.05. Multivariable Cox regression model of survival was used to estimate the proportional hazard ratio and 95% CI to be diagnosed with TB. As potential influencing factors, we incorporated clinical covariates (ART, viral load, and CD4 cell count at the start of observation), as well as epidemiological factors such as sex, HIV transmission risk group, and origin. Concerning the latter, we conducted two analyses, one based on the geographical region of origin and another based on the TB incidence of the country of origin. No time-varying variables were utilized in the analyses. TB incidence density rates (IDR) for different groups and time spans were compared and tested for statistical significance using MedCalc’s Free statistical calculators (https://www.medcalc.org/calc/index.php, MedCalc Software, Ostend, Belgium). TB IDR was defined by the number of TB cases occurring per 100 patient-years of observation. IDR calculations were performed for the entire cohort as well as for specific groups within the cohort, delineated by clinical and demographic characteristics. We defined three periods during the observation span to describe IDR trends over time, 2006–2009, 2010–2013, and 2014–2017. These periods reflect changes in the overall TB incidence in Germany—sharply declining case numbers in the years 2006–2009, relatively stable numbers of cases in the years 2010–2013, and the significant increase in 2014 to 2016 [13]. Furthermore we calculated and analyzed the development of TB IDR depending on time since ART was initiated in patients. For these calculations, only patients who started ART in 1996 (year when first protease inhibitors became available) or later with an observation time of at least 90 days after therapy start were included. In addition, TB cases that occurred within 90 days of starting ART were excluded, as unmasking of pre-existing TB by immune reconstitution could not be ruled out, similar to the approach of Karo et al. [17].

### Ethical considerations

The study was approved by the Ethical Committee of the Medical Faculty of the University of Cologne (13-364) and Bonn (279/14). All patients enrolled in the Cologne/Bonn HIV cohort provided written informed consent prior to inclusion.

## Results

### Patients’ characteristics and outcome

**Fig. 1:**
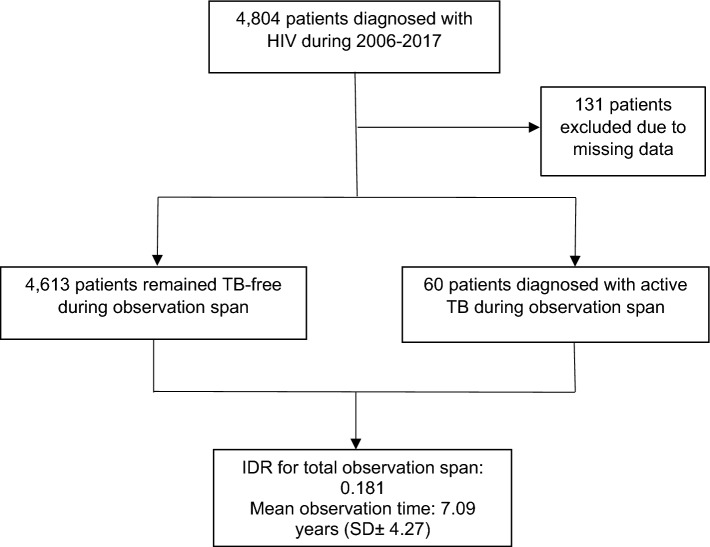
Outline of study design and findings regarding incidence density rate (IDR). *TB* tuberculosis, *IDR* incidence density rate (cases per 100 patient-years of observation), *SD* standard deviation

A total of 4673 patients from the Cologne/Bonn HIV cohort, enrolled between 2006 and 2017, contributed to the dataset (Fig. 1).

On average, 1830 [1616–1907] patients from this cohort had annual visits in Cologne and 1115 [1042–1148] in Bonn. Patient characteristics, demographic and clinical data are shown in Table 1.

**Table 1:** Demographic and clinical characteristics of people living with HIV/AIDS or with HIV/TB coinfection in the Cologne/Bonn cohort, 2006–2017

Characteristics	Non-tuberculosis *N* = 4613	Tuberculosis *N* = 60	*p* value	Total *N* = 4673
Sex			< 0.001	
Male	3705 (80.3)	37 (61.7)		3742 (80.1)
Female	904 (19.6)	23 (38.3)		927 (19.8)
Missing				4 (0.1)
Death	293 (6.4)	6 (10)	0.526	299 (6.4)
Age at start of observation^a^			0.013	
≤ 38 years	2026 (43.9)	36 (60)		2062 (44.1)
> 38 years	2587 (56.1)	24 (40)		2611 (55.9)
Age at the time of TB diagnosis, years	–	38.6 [31.9–45.4]		–
Time between migration to Germany and TB diagnosis				
< 1 year	–	5 (8.3)		–
1–5 years	–	10 (16.7)		–
5–10 years	–	2 (3.3)		–
10–15 years	–	1 (1.7)		–
15–19 years	–	3 (5)		–
≥ 20 years	–	4 (6.7)		–
TB before migration	–	5 (8.3)		–
No migration	–	13 (21.7)		–
Missing	–	17 (28.3)		–
Region of origin				
Germany	3369 (73.0)	14 (23.3)	*	3383 (72.4)
Sub-Sahara Africa	417 (9.0)	22 (36.7)	< 0.001; *	439 (9.4)
Other origin/unknown	827 (17.9)	24 (40.0)	< 0.001; 0.04	851 (18.2)
TB incidence of country of origin				
< 10/100,000	3380 (73.3)	16 (26.7)	*	3396 (72.7)
10–50/100,000	322 (7)	12 (20)	< 0.001; *	334 (7.2)
> 50/100,000	501 (10.9)	32 (53.3)	< 0.001; 0.13	533 (15.1)
Missing				410 (8.8)
HIV transmission risk group				
MSM	2421 (52.5)	13 (21.7)	*	2434 (52.1)
Heterosexual contacts	909 (19.7)	22 (36.7)	< 0.001	931 (19.9)
PWID	288 (6.2)	2 (3.3)	0.740	290 (6.2)
ICGE	394 (8.5)	13 (21.7)	< 0.001	407 (8.7)
Other (e.g., blood products)	173 (3.8)	1 (1.7)	0.940	174 (3.7)
Unknown	415 (9.0)	8 (13.3)		423 (9.1)
Missing				14 (0.3)
CD4 + cell count at start of observation^a^, cells/µl	387 [220–570]	230 [110–420]	< 0.001	382 [218–570]
Missing	364	3		367
CD4 + cell count at the time of tuberculosis diagnosis, cells/µl	–	150 [68–300]		–
Missing		10		
Viral load at start of observation^a^, copies/ml	3316 [49–58,166]	60,433 [260–184,051]	< 0.001	3390 [49–59,257]
Missing	118	6		124
Viral load at the time of tuberculosis diagnosis, copies/ml	–	76,906 [8981–514,036]		–
Patients with VL < 50 copies/ml		5		
Missing		12		
Antiretroviral therapy	4059 (88.0)	25 (41.7)^b^	< 0.001	4084 (87.4)

Number (percentage), median [interquartile range]. Statistical comparison between tuberculosis and non-tuberculosis group using *χ*^2^-test for categorical variables and Mann–Whitney *U* test for continuous variables; significance level of 5% for both tests

*MSM* men who have sex with men, *PWID* people who inject drugs, *ICGE* immigrants from countries with generalized HIV epidemic, *VL* viral load

*Reference group for statistical comparison

^a^Start of observation defined as HIV initial diagnosis or 01.01.2006 if initial diagnosis took place before the observation period

^b^At any time before diagnosis of tuberculosis

Patients were predominantly male (3742, 80%) and of German origin (3383, 72%), i.e., born in Germany. The second most prevalent region of origin was Sub-Saharan Africa (439, 9%). In the entire cohort, median age of females at initial diagnosis of HIV was significantly lower compared to males (30.52 years vs. 35.16 years respectively, *p* < 0.001). The predominant HIV transmission risk group was men who have sex with men (MSM) in male patients (2434, 65%) and heterosexual contacts in female patients (436, 47%).

During the observation period, 60 (37 male, 23 female) patients were diagnosed with active TB. People from Sub-Saharan Africa accounted for a relevant group with 37% (22/60) whereas only 23% of all HIV/TB-coinfected individuals originated from Germany (Table 1). The remaining proportion (24/60, 40%) originated from other countries or regions. There was a significantly different proportion of individuals originating from other countries than Germany for coinfected women and men, with 91% (21/23) being female and 68% (25/37) being male (*p* = 0.035). A large part (16/23, 70%) of the female patients originated from Sub-Saharan Africa, while Germany was the main origin among male patients (12/37, 32%).

For HIV/TB-coinfected patients, the median age at time of initial HIV diagnosis was 35.1 years (IQR 28.1–39.6) whereas median age at initial TB diagnosis was 38.6 (IQR 31.9–45.4) years. In patients originating from Germany, active TB was more likely to occur in people > 38 years of age. In HIV/TB-coinfected individuals from foreign countries, TB was predominately among patients < 38 years of age (24/46, 52%).

Women were significantly younger than men at time of TB diagnosis (35.6 years [IQR 30.5–40.6], 41.2 years [IQR 34.6–48.2] respectively, *p* = 0.049).

Overall, 22 of the 60 coinfected patients (37%) were diagnosed concomitantly for HIV and active TB.

In the entire cohort, 87.4% (4084/4673) were treated with ART, whereas only 41.7% (25/60) of the HIV/TB-coinfected patients had received ART prior to their TB diagnosis (*p* < 0.001). At the beginning of the observation period, median CD4 + cell counts were significantly lower among HIV/TB-coinfected individuals compared to the non-tuberculosis group (230 cells/µl [110–420] vs. 387 cells/µl [220–570], respectively, *p* < 0.001). The median viral load (VL) was significantly higher (60,433 copies/ml [259–184,052] vs. 3316 [49–58,166], *p* < 0.001) in HIV/TB-coinfected patients (Table 1). In the 60 patients with HIV/TB coinfection, isolated pulmonary TB accounted for 35% (21/60), followed closely by disseminated TB (infection involving 2 or more non-contiguous sites) at 31.7% (19/60). Isolated lymph node tuberculosis represented the third most common entity in our cohort, comprising 16.7% (10/60) of cases (Table 2).

**Table 2:** Tuberculosis manifestation in the Cologne/Bonn cohort, 2006–2017

Body region of manifestation *N* = *60*	
Lung	21 (35)
Lymph nodes	10 (16.7)
Pleura	1 (1.7)
Bones	1 (1.7)
Digestive tract	3 (5)
Disseminated	19 (31.7)
Meninges	2 (3.3)
Missing	3 (5)

Number (percentage)

### TB-free survival

TB-free overall survival was 98.7% throughout the entire cohort, with significant differences regarding sex, origin, status of ART, CD4 + cell count, and viral load at observation start. Women had a lower TB-free survival compared to men, especially when ART had not been initiated before the time of TB diagnosis, with only 87.6% survival rate for women vs. 95.6% for men (*p* = 0.002). CD4 + cell count < 200/µl and viral load > 5 log_10_ copies/ml had a negative impact on TB-free survival rates (*p* < 0.001), particularly among patients who never started ART (Supplemental Table 1).

**Fig. 2:**
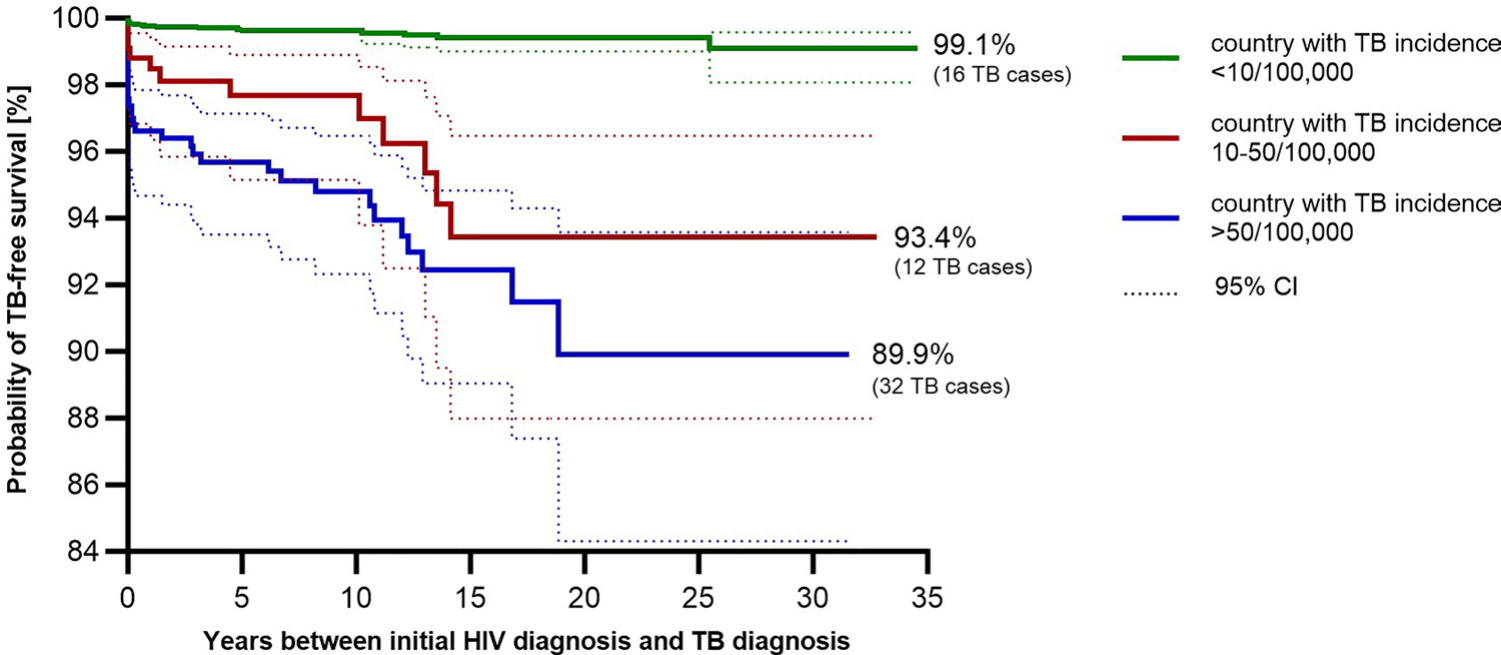
TB-free survival of patients with known country of origin (4263/4673, 91.2%) based on TB incidences in the countries of origin according to the 2023 WHO Global Tuberculosis Report. The incidences were divided into three groups: < 10/100,000 (*n* = 3396), 10–50/100,000 (*n* = 334), and > 50/100,000 (*n* = 533). In the statistical analysis (log-rank test; significance level 5%), the curves show a significant difference (*p* < 0.001)

Patients originating from Sub-Saharan Africa showed the lowest TB-free survival rates compared to patients originating from Germany (95.0 vs. 99.6%, *p* < 0.001) or other countries or regions (97.2%). The TB-free survival rate for individuals originating from Sub-Saharan Africa dropped further (75.0%) if ART had not been initiated at the time of TB diagnosis (in comparison to 98.2% of German patients, *p* < 0.001). Patients originating from Germany, on ART, and with CD4 + cell counts ≥ 200 cells/µl had the best outcome (TB-free survival 99.9%) while lowest TB-free survival rate (16.7% survival rate; *p* = 0.001) was seen among patients without ART, originating from Sub-Saharan Africa and with a CD4 + cell count < 200 cells/µl. Overall, we observed that patients originating from countries with a TB incidence ≥ 10–50/100,000 and > 50/100,000 showed significantly lower TB-free survival (93.4% and 89.9%) than patients originating from countries with a TB incidence < 10/100,000 (99,1%, *p* < 0.001) (Fig. 2).

Multivariable Cox regression models to identify associated risk factors for TB confirmed the Kaplan–Meier data described above. We included sex, region of origin or the origin stratified by TB incidence, respectively, as well as ART status, CD4 + cell count, and viral load at enrollment as influencing factors. The adjusted hazard ratios (HR) for the association of factors are presented in Supplemental Table 2. Patients originating from countries other than Germany, particularly Sub-Saharan African countries, or from countries with TB incidences > 10/100,000, and patients with CD4 + cell counts below 200 cells/µl were significantly more likely to develop TB. In this evaluation, a high viral load (≥ 5 log_10_ copies/ml) indicated a higher risk which was not statistically significant. The most influential factor was the absence of ART before the diagnosis of TB, leading to a more than tenfold increased risk for active TB. Patients on ART had the highest hazard ratio for developing TB if they originated from Sub-Saharan Africa (HR 13.42 CI [3.59–50.13], *p* < 0.001) or a country with a TB incidence > 50/100,00 (HR 8.04 CI [2.76–23.45], *p* < 0.001) (Supplemental Table 2).

### TB incidence density rate (IDR) during the observation period

To better compare our data to previously published studies, we determined IDR values. There was a total of 33,136.14 years of observation for 4673 patients. With a mean observation time of 7.09 years (± 4.27 SD), the IDR for the entire observation span was 0.181 cases per 100 patient-years of observation (CI [0.138–0.233]) (Table 3).

**Table 3:** Incidence density rate (IDR) stratified by period

Period	Sum patient-years	TB cases	IDR [95%]
01.01.2006–31.12.2009	10,513.64	28	0.266 [0.177–0.385]
01.01.2010–31.12.2013	11,351.38	17	0.150 [0.087–0.240]
01.01.2014–31.12.2017	11,271.12	15	0.133 [0.075–0.220]
Total	33,136.14	60	0.181 [0.138–0.233]

IDR, incidence density rate (cases per 100 patient-years of observation); [95%], 95% confidence interval

As expected, a significantly higher TB IDR was found for patients originating from Sub-Saharan Africa (0.694 cases per 100 patient-years of observation, CI [0.435–1.050] *p* = < 0.001) or from countries with a TB incidence > 10/100,000 (Table 4). Also in female patients (0.342 cases per 100 patient-years of observation, CI [0.217–0.513], *p* = 0.0005), patients with CD4 + cell counts < 200 cells/µl (0.444 cases per 100 patient-years of observation, CI [0.290–0.651], *p* < 0.001) and viral loads ≥ 5 log_10_ (0.428 cases per 100 patient-years of observation, CI [0.262–0.662], *p* < 0.001) showed significantly higher TB IDRs (Table 4).

**Table 4:** TB incidence density rate (IDR) stratified by demographic and clinical characteristics at enrollment, Cologne–Bonn cohort, initial HIV diagnosis between 2006 and 2017

Characteristics	No. of patients	Patient-years	No. with TB	TB IDR [95% CI]	*p* value
Total patients	4673	33,136.14	60	0.181 [0.138–0.233]	
**Sex**					< 0.001
Male	3742	26,387.85	37	0.140 [0.099–0.193]	
Female	927	6724.45	23	0.342 [0.217–0.513]	
Missing	4	–	–	–	
**Age at start of observation**					0.005
≤ 38	2062	13,910.20	36	0.259 [0.181–0.358]	
> 38	2611	19,193.94	24	0.125 [0.080–0.186]	
**Region of origin**					
Germany	3383	24,411.18	13	0.053 [0.028–0.091]	
Sub-Sahara Africa	439	3171.15	22	0.694 [0.435–1.050]	< 0.001
Other countries	851	5553.82	25	0.450 [0.291–0.665]	< 0.001
**TB incidence of country of origin**					
< 10/100,000	3396	24,275.87	16	0.066 [0.038–0.107]	
10–50/100,000	334	2213.14	12	0.542 [0.280–0.947]	< 0.001
> 50/100,000	533	3724.03	32	0.859 [0.588–1.213]	< 0.001
Missing	410	–	–	–	
**HIV transmission risk group**					
MSM	2434	17,041.62	13	0.076 [0.041–0.130]	
Heterosexual contacts	931	6494.01	21	0.323 [0.200–0.494]	< 0.001
PWID	290	1975.27	1	0.051 [0.001–0.282]	0.691
ICGE	407	3196.69	13	0.407 [0.217–0.695]	< 0.001
**CD4 + cell count (cells/µl)**^a^					
> 500	1393	10,954.55	10	0.091 [0.044–0.168]	
351–500	994	7630.40	6	0.079 [0.029–0.171]	0.773
200–350	929	6673.30	15	0.225 [0.126–0.371]	0.022
< 200	990	5854.60	26	0.444 [0.290–0.651]	< 0.001
Missing	367	–	–	–	
**Viral load (log10 copies/ml)**^a^					< 0.001
< 5	3670	28,183.17	34	0.121 [0.084–0.169]	
≥ 5	879	4668.96	20	0.428 [0.262–0.662]	
Missing	124	–	–	–	
**Antiretroviral therapy**^b^					< 0.001
*Never started*	589	2297.64	35	1.523 [1.061–2.118]	
Germany	390	1523.09	7	0.460 [0.185–0.947]	
Sub-Sahara Africa	48	215.35	12	5.581 [2.994–9.750]	
Other countries	135	559.2	16	2.862 [1.636–4.648]	
< 10/100,000^c^	370	1476.29	6	0.406 [0.149–0.885]	
10–50/100,000^c^	42	157.39	9	5.732 [2.621–10.882]	
> 50/100,000^c^	74	269.56	20	7.407 [4.525–11.440]	
*Under treatment*	4084	30,838.49	25	0.081 [0.052–0.120]	
Germany	2993	22,888.09	7	0.031 [0.012–0.063]	
Sub-Sahara Africa	391	2955.77	10	0.338 [0.162–0.622]	
Other countries	700	4994.62	8	0.160 [0.069–0.316]	
< 10/100,000^c^	3026	23,180.87	10	0.043 [0.021–0.079]	
10–50/100,000^c^	292	2056.02	3	0.146 [0.030–0.426]	
> 50/100,000^c^	459	3454.47	12	0.347 [0.180–0.607]	

Comparison of IDR by analyzing IDR differences; statistical testing using *χ*^2^-test; significance level 5%

*IDR* incidence density rate (cases per 100 patient-years of observation), *MSM* men who have sex with men, *PWID* people who inject drugs, *ICGE* immigrants from countries with generalized HIV epidemic

^a^At start of observation, defined as HIV initial diagnosis or 01.01.2006 if initial diagnosis took place before the observation period

^b^At any time before TB diagnosis

^c^TB incidence of country of origin

There was an approximately 10- to 20-fold higher TB IDR among patients who did not receive ART (1.523 cases per 100 patient-years of observation, CI [1.061–2.118], *p* < 0.001). This ratio also remained after analysis by region and TB incidence of origin (Table 4).

TB IDR was increasing with decreasing CD4 + cell count, this trend was constantly observed in each subgroup. The highest TB IDR was found among patients who did not receive ART and patients with a CD4 + cell count below 200 cells/µl (16.850 cases per 100 patient-years of observation, CI [9.430–27.800]).

### Longitudinal trend in TB/HIV coinfection

A comparison of consecutive 4-year periods during the observation time span indicated a decline in TB IDR in our cohort (Table 3). Between 2006–2009, the TB IDR ranked at 0.266 cases per 100 patient-years of observation (CI [0.177–0.385]), while it decreased non-significantly to 0.150 cases per 100 patient-years of observation (CI [0.087–0.240], *p* = 0.057) during 2010–2013 and further to 0.133 cases per 100 patient-years of observation (CI [0.075–0.220]) during 2014–2017(*p* = 0.739). Comparing the values of the earliest period with the latest, the decline was significant (*p* = 0.0270). This drop in IDR basically remained when stratifying by date of initial HIV diagnosis before or after 01st of January 2006. The IDRs for the aforementioned time periods were 0.158, 0.123, and 0.066 cases per 100 patient-years of observation for patients with initial HIV diagnosis before, and 0.896, 0.217, 0.240 cases per 100 patient-years of observation for patients with initial HIV diagnosis after 1st of January 2006 (Supplemental Table 3). Individuals on ART had lower IDR values than the overall cohort in all three periods and also showed the trend of decreasing IDR over time, although the proportion of individuals with a suppressed viral load at enrollment also decreased over time (Supplemental Table 3). Overall, the highest TB IDR in the entire cohort was 0.421 cases per 100 patient-years of observation in 2007.

Looking at the IDR as a function of time after the start of antiretroviral therapy, there was a significant decrease up to 7 years after the start of ART (*p* = 0.0001). Three years after the start of ART, the IDR had already been reduced by more than 75% compared to that of the first year (0.141 CI [0.046–0.33] vs. 0.033 CI [0.007–0.097]; *p* = 0.03) (Supplemental Fig. 1).

## Discussion

In this work, we longitudinally determined the incidence of HIV/TB coinfections in a large HIV patient cohort of two German University Hospitals. In addition, statistical analyses were conducted to identify risk factors associated with active TB in PLWH.

In Europe, data on the HIV status of TB patients were available in about 70% of all cases [2, 4]. Among the 21 European countries with documented HIV status, approximately 4% of all TB patients were HIV positive [4]. These numbers have remained stable in the past years [2–4, 18, 19]. Germany is among the countries in which precise epidemiological data on HIV/TB coinfections are not available [13]. However, it is estimated that the proportion of active TB among HIV-positive patients is approximately 2.6% (1.4–4.1%) [2].

In our study, we calculated the IDR, a measure that incorporates time as a parameter in the denominator. This approach is considered more accurate for estimating disease risk within a dynamic cohort [20]. The IDR we determined across the cohort was 0.181 cases per hundred patient-years. A decrease in the IDR could be seen over the entire time span with a decrease of 50% over all 4-year periods (0.266–0.133 cases per 100 patient-years of observation). Another German study found a TB IDR in PLWH of 0.37 [21]. The higher IDR observed in this study may be explained by the earlier observation period ranging from 2001 to 2011. Notably, in our study, the highest IDR for a single year was 0.421 cases per 100 patient-years of observation in 2007, a year which was also covered by the study of Karo et al. Interestingly, in our study, the decline of HIV/TB coinfected patients and a relatively low IDR in the 2014 to 2017 period was observed despite a strong increase of the overall TB incidence in Germany in 2015 and 2016 due to the conflict in Syria and the subsequent migration flow to central Europe [22]. As German guidelines advocate HIV testing for all patients diagnosed with active TB, we assume that the overall HIV incidence in these TB-positive migrants, many of whom were refugees, was low.

We observed a significant increase in the IDR based on country or region of origin, with an almost fivefold higher IDR in patients originating from Sub-Saharan Africa. Furthermore, patients with signs of advanced and untreated HIV (CD4 cell count < 200/µl and high viral load > 5 log) showed an increased TB rate. In line, we found an up to 20-fold increase in IDR in patients without ART compared to patients treated with ART. Conversely, we were able to show that the IDR fell significantly with increasing duration of ART. The combination of these factors further elevated the IDR, with the highest IDR observed in patients with low CD4 + cell counts not yet on ART. Consequently, we observed that HIV infection was often diagnosed concomitantly with the TB diagnosis in individual patients. It is noteworthy that in our cohort, the coinfection of HIV and TB frequently led to severe disease, as evidenced by the high rate of disseminated TB (31.7%). An assessment of our data using Kaplan–Meier survival analyses or Cox regression analyses yielded consistent results regarding risk factors for active TB in PLWH. Overall, our observations are in line with findings made in other studies investigation HIV/TB coinfections in low-incidence countries [17, 21, 23–25].

With regard to a correlation between HIV transmission risk groups and the diagnosis of TB, a significant risk was found in patients with probable heterosexual acquisition of the HIV infection. This observation may be influenced by the patients’ origin: while in Western Europe and North America, HIV infection tends to occur through male homosexual contacts, heterosexual contacts represent the most frequent HIV transmission route in African countries, which also exhibit the highest TB incidences. This may also explain an increased risk of developing active TB in women originating from Sub-Saharan Africa. A finding which is in line with data from other studies [26–30].

According to the HIV/TB risk factors and the relatively low and declining IDR, we identified in our cohort, our data align with the recent recommendations in German guidelines, recommending risk-adapted screening for latent TB in HIV-infected individuals [11]. While older recommendations suggested interferon-gamma release assay (IGRA)-based testing in all HIV-positive individuals, current recommendations take the immune status, place of origin, and ART status into account [9, 11]. However, it is important to note that latent TB was not assessed in our study, and the development of active TB over time serves as a surrogate for the number of latently infected patients in a specific cohort.

### Limitations

Our study has several limitations. The observation period ended in 2017 and lacks an evaluation of the past 6 years. It is possible that the epidemiological situation in Germany changed considerably at later time points. Notably, the data presented here remain unaffected by the SARS-CoV-2 pandemic, a period during which numerous diseases, including HIV and TB, were potentially underdiagnosed [12, 31–33]. In addition, migration to Germany was reduced by global lockdowns. It is, therefore, likely that this period would have resulted in a very low IDR, which may not accurately reflect the actual epidemiological situation. Given the influence of migration from middle- or high-incidence countries on TB incidence in Germany, it can be assumed that the incidence of TB/HIV coinfection will also be affected by the war in Ukraine and the associated increased migration of Ukrainians toward Western Europe. Ukraine stands among Europe’s countries with the highest incidences of HIV and TB [3, 12, 34]. Hence, it is crucial to continuously observe epidemiological data and adapt guidelines for screening of patients at risk for both diseases.

## Conclusion

This study provides further evidence that HIV patients originating from regions with high TB incidence bear a higher risk of falling ill with active TB in Germany. In contrast, for PLWH born in Germany, the risk of active TB is lower which supports the decision of risk-adapted TB screening strategies for PLWH in low-incidence countries. In addition, HIV remains a risk factor for severe forms of TB such as disseminated disease even in low-incidence countries. We found that, in several patients, both diseases were diagnosed simultaneously. Thus, the clinical workup of newly diagnosed TB and HIV patients requires increased attention of physicians to avoid diagnostic delay of the respective coinfecting disease and the subsequent care of patients in specialized centers.

### Supplementary Information

Below is the link to the electronic supplementary material.

Supplementary file1 (DOCX 122 KB)

## Data Availability

The datasets generated and/or analysed during the current study are not publicly available due to ethical and legal restrictions but are available from the corresponding author upon reasonable request.

## References

[1] ECDC/WHO. Tuberculosis surveillance and monitoring report in Europe 2019. 2019.

[2] ECDC/WHO. Tuberculosis surveillance and monitoring in Europe 2022. 2022.

[3] WHO. Global tuberculosis report 2023. 2023.

[4] ECDC/WHO. Tuberculosis surveillance and monitoring in Europe 2023. 2023.

[5] UNAIDS. Fact sheet—world tuberculosis day 2022. 2022.

[6] Getahun H, et al. HIV infection-associated tuberculosis: the epidemiology and the response. Clin Infect Dis. 2010;50:S201–7. 20397949 10.1086/651492

[7] WHO. Global tuberculosis report 2019. 2019.

[8] Tiberi S, et al. The cursed duet today: tuberculosis and HIV-coinfection. Presse Med. 2017;46:e23–39. 28256380 10.1016/j.lpm.2017.01.017

[9] Schaberg T, et al. Tuberculosis guideline for adults—guideline for diagnosis and treatment of tuberculosis including LTBI testing and treatment of the German Central Committee (DZK) and the German Respiratory Society (DGP). Pneumologie. 2017;71:325–97. 28651293 10.1055/s-0043-105954

[10] WHO. Guidelines for treatment of drug-susceptible tuberculosis and patient care, 2017 update. 2017.

[11] Schaberg T, et al. Tuberculosis in adulthood—the Sk2-guideline of the German Central Committee against Tuberculosis (DZK) and the German Respiratory Society (DGP) for the diagnosis and treatment of adult tuberculosis patients. Pneumologie. 2022;76:727–819. 36384164 10.1055/a-1934-8303

[12] WHO. Global tuberculosis report 2022. 2022.

[13] Robert-Koch-Institut. Bericht zur Epidemiologie der Tuberkulose in Deutschland 2022. 2023.

[14] Bundesamt S. Migration 2021: 329 000 Personen mehr zu- als abgewandert. 2022.

[15] Rockstroh JK. The Cologne–Bonn cohort: lessons learned. Infection. 2015;43:135–9. 25708018 10.1007/s15010-015-0745-2

[16] Ehren K, et al. Causes of death in HIV-infected patients from the Cologne–Bonn cohort. Infection. 2014;42:135–40. 24081925 10.1007/s15010-013-0535-7

[17] Karo B, et al. Immunological recovery in tuberculosis/HIV co-infected patients on antiretroviral therapy: implication for tuberculosis preventive therapy. BMC Infect Dis. 2017;17:517. 28743248 10.1186/s12879-017-2627-y PMC5526303

[18] ECDC/WHO. Tuberculosis surveillance and monitoring in Europe 2017. 2017.

[19] Aldridge RW, et al. Tuberculosis in migrants moving from high-incidence to low-incidence countries: a population-based cohort study of 519 955 migrants screened before entry to England, Wales, and Northern Ireland. Lancet. 2016;388:2510–8. 27742165 10.1016/S0140-6736(16)31008-X PMC5121129

[20] CDC. Principles of epidemiology/lesson 3: measures of risk. 2012.

[21] Karo B, et al. Tuberculosis among people living with HIV/AIDS in the German ClinSurv HIV Cohort: long-term incidence and risk factors. BMC Infect Dis. 2014;14:148. 24646042 10.1186/1471-2334-14-148 PMC3994660

[22] Robert-Koch-Institut. Bericht zur Epidemiologie der Tuberkulose in Deutschland für 2015. 2016

[23] Sonnenberg P, et al. How soon after infection with HIV does the risk of tuberculosis start to increase? A retrospective cohort study in South African gold miners. J Infect Dis. 2005;191:150–8. 15609223 10.1086/426827

[24] Bruchfeld J, Correia-Neves M, Kallenius G. Tuberculosis and HIV Coinfection. Cold Spring Harb Perspect Med. 2015;5: a017871. 25722472 10.1101/cshperspect.a017871 PMC4484961

[25] Bell LCK, Noursadeghi M. Pathogenesis of HIV-1 and *Mycobacterium tuberculosis* co-infection. Nat Rev Microbiol. 2018;16:80–90. 29109555 10.1038/nrmicro.2017.128

[26] Kwan CK, Ernst JD. HIV and tuberculosis: a deadly human syndemic. Clin Microbiol Rev. 2011;24:351–76. 21482729 10.1128/CMR.00042-10 PMC3122491

[27] Seneadza NAH, et al. Assessing risk factors for latent and active tuberculosis among persons living with HIV in Florida: a comparison of self-reports and medical records. PLoS ONE. 2022;17:e0271917. 35925972 10.1371/journal.pone.0271917 PMC9352085

[28] Adhikari N, et al. Prevalence and associated risk factors for tuberculosis among people living with HIV in Nepal. PLoS ONE. 2022;17: e0262720. 35089953 10.1371/journal.pone.0262720 PMC8797228

[29] Aung ZZ, et al. Survival rate and mortality risk factors among TB-HIV co-infected patients at an HIV-specialist hospital in Myanmar: a 12-year retrospective follow-up study. Int J Infect Dis. 2019;80:10–5. 30572021 10.1016/j.ijid.2018.12.008

[30] Shah GH, et al. Risk factors for TB/HIV coinfection and consequences for patient outcomes: evidence from 241 clinics in the Democratic Republic of Congo. Int J Environ Res Public Health. 2021;18:5165. 34068099 10.3390/ijerph18105165 PMC8152772

[31] Dinmohamed AG, et al. Fewer cancer diagnoses during the COVID-19 epidemic in the Netherlands. Lancet Oncol. 2020;21:750–1. 32359403 10.1016/S1470-2045(20)30265-5 PMC7252180

[32] Del Cura-Gonzalez I, et al. What have we missed because of COVID-19? Missed diagnoses and delayed follow-ups. SESPAS Report 2022. Gac Sanit. 2022;36:S36–43. 35781146 10.1016/j.gaceta.2022.03.003 PMC9244613

[33] Visca D, et al. Tuberculosis and COVID-19 interaction: a review of biological, clinical and public health effects. Pulmonology. 2021;27:151–65. 33547029 10.1016/j.pulmoe.2020.12.012 PMC7825946

[34] ECDC/WHO. HIV/AIDS surveillance in Europe 2021 (2020 data). 2021.

