# Supplementary material for: Incidence and risk factors for HIV-tuberculosis coinfection in the Cologne–Bonn region: a retrospective cohort study
Source: Infection. 2024 Mar 16;52(4):1439–48. doi: 10.1007/s15010-024-02215-y (PMC11289312; doi:10.1007/s15010-024-02215-y)
Supplement: Supplementary file 1 — Supplementary file1 (DOCX 122 KB) [file 15010_2024_2215_MOESM1_ESM.docx]

**Supplement Information**

**Incidence and risk factors for HIV-tuberculosis coinfection in the Cologne-Bonn region, a retrospective cohort study**

Isabelle Suárez*^1,2^, Dominic Rauschning*^1,7^, Cora Schüller^1^, Anna Hagemeier^5^, Melanie Stecher^1,2^, Clara Lehmann^1,2,3^, Philipp Schommers^1,2,3^, Stefan Schlabe^4^, Jörg-Janne Vehreschild^1,2,6^, Carolin Koll^1^, Carolynne Schwarze-Zander^2,4,8^, Jan-Christian Wasmuth^4^, Angela Klingmüller ^1^, Jürgen Kurt Rockstroh^2,4^, Gerd Fätkenheuer^1,2^ , Christoph Boesecke^4^, Jan Rybniker^1,2,3^

1. Department I of Internal Medicine, Medical Faculty and University Hospital Cologne, University of Cologne, 50937 Cologne, Germany
2. German Center for Infection Research (DZIF), Partner Site Bonn-Cologne, Cologne, Germany
3. University of Cologne, Center for Molecular Medicine Cologne, Cologne, Germany
4. Department of Medicine I, University Hospital Bonn, Bonn, Germany
5. Institute of Medical Statistics and Computational Biology, Medical Faculty and University Hospital Cologne, University of Cologne, Cologne, Germany
6. Goethe University Frankfurt, University Hospital, Center for Internal Medicine, Medical Department 2 (Hematology/Oncology and Infectious Diseases), Frankfurt am Main, Germany
7. Bundeswehrzentralkrankenhaus Koblenz, Department IB of Internal Medicine, Koblenz, Germany
8. Gemeinschaftspraxis am Kaiserplatz, Bonn, Germany

*contributed equally

**Corresponding Author:**

Jan Rybniker, M.D., PhD

Department I of Internal Medicine
University Hospital of Cologne

Kerpener Str. 62

50937 Cologne, Germany

Tel.: 0049-221 478-89611

Email: [jan.rybniker@uk-koeln.de](mailto:jan.rybniker@uk-koeln.de)

**Supplemental Tables**

| **Characteristics** | **Total** | | **Never received ART** | | **Received**  **ART^1^** | | **Origin Germany** | | **Origin Sub-Sahara Africa** | | **Origin**  **Other** | |
| --- | --- | --- | --- | --- | --- | --- | --- | --- | --- | --- | --- | --- |
|  | Survival rates (%) | *p-*  *value* | Survival rates (%) | *p-*  *value* | Survival rates (%) | *p-*  *value* | Survival rates (%) | *p-*  *value* | Survival rates (%) | *p-*  *value* | Survival rates (%) | *p-*  *value* |
| **Total** | **98.7** | *-* | **94.1** | *-* | **99.4** | *-* | **99.6** | *-* | **95.0** | *-* | **97.2** | *-* |
| **Sex**    Male    Female | **99.0**  **97.5** | *<0.001* | 95.6  87.6 | *0.002* | 99.5  98.9 | *0.045* | 99.6  99.5 | *0.826* | 96.0  94.4 | *0.459* | 97.0  97.8 | *0.486* |
| **Origin**    Germany    Sub-  Sahara Africa    Other countries | **99.6**  **95.0**  **97.2** | *<0.001* | 98.2  75.0  89.4 | *<0.001* | 99.8  97.4  98.9 | *<0.001* | - | *-* | - | *-* | - | *-* |
| **CD4+ cell count^2^**    ≥200    <200 | **99.1**  **97.4** | *<0.001* | 96.3  68.1 | *<0.001* | 99.6  98.8 | *0.006* | 99.8  98.8 | *<0.001* | 96.0  92.8 | *0.114* | 97.6  95.8 | *0.139* |
| **VL copies/ml^2^**    <5log10    ≥5log10 | **99.1**  **97.7** | *<0.001* | 95.6  88.3 | *0.001* | 99.5  98.9 | *0.010* | 99.7  99.1 | *0.010* | 96.8  90.2 | *0.003* | 97.5  95.6 | *0.131* |
| **Age at observation start^2^**    >38    ≤38 | **99.1**  **98.3** | *0.009* | 94.9  93.4 | *0.424* | 99.5  99.2 | *0.155* | 99.7  99.5 | *0.414* | 95.9  94.4 | *0.400* | 97.5  96.9 | *0.574* |
| **Age at HIV ID**    >38    ≤38 | **99.0**  **98.5** | *0.197* | 94.9  93.7 | *0.873* | 99.5  99.3 | *0.345* | 99.6  99.6 | *0.726* | 95.9  94.7 | *0.623* | 97.5  97.0 | *0.784* |
| **HIV-transmission risk group**    MSM  HC    PWID    ICGE | **99.5**  **97.6**  **99.3**  **96.8** | *<0.001* | 97.5  86.4  98.0  77.4 | *<0.001* | 99.7  99.0  99.6  98.4 | *0.062* | - | *-* | - | *-* | - | *-* |
| **ART**    Never received    Received | **94.5**  **99.4** | *<0.001* | - | *-* | - | *-* | 98.2  99.8 | *<0.001* | 75.0  97.4 | *<0.001* | 89.4  98.9 | *<0.001* |

^1^, at any time before diagnosis of tuberculosis; ^2^, start of observation being defined as 01.01.2006 or HIV initial diagnosis date if later than 01.01.2006

ART, antiretroviral therapy; VL, viral load; HIV ID, HIV initial diagnosis; MSM, men who have sex with men; HC, heterosexual contacts; PWID, people who inject drugs; ICGE, immigrants from countries with generalised HIV epidemic

Statistical comparison of survival rates by using log-rank-test; significance level 5%.

**Supplemental Table 1:** Tuberculosis free survival in the Cologne/Bonn Cohort by demographic and clinical characteristics, 2006-2017

**A**

| \| **Predictor/risk variable for falling ill with tuberculosis** \| **Total patients** \| \| **Patients never started ART** \| \| **Patients on ART^1^** \| \| **Germany** \| \| **Sub-Sahara Africa** \| \| **Other countries** \| \| \| --- \| --- \| --- \| --- \| --- \| --- \| --- \| --- \| --- \| --- \| --- \| --- \| --- \| \| HR [95%] \| *p-*  value \| HR [95%] \| *p-*  value \| HR [95%] \| *p-*  value \| HR [95%] \| *p-*  value \| HR [95%] \| *p-*  value \| HR [95%] \| *p*-  value \| \| **Sex**  Female  Male \| 1  0.98 [0.51-1.88] \| 0.957 \| 1  0.78 [0.33-1.86] \| 0.576 \| 1  1.25  [0.46-3.40] \| 0.663 \| 1  1.56 [0.30-8.01] \| 0.596 \| 1  0.71  [0.25-2.00] \| 0.510 \| 1  1.15  [0.36-3.68] \| 0.821 \| \| **Region of Origin**  Germany  Sub-Sahara Africa    Other  countries \| 1  7.65 [3.19-18.32]  5.14 [2.49-10.62] \| <0.001    <0.001 \| 1  5.54 [1.62-18.99]  5.03 [1.79-14.19] \| 0.006    0.002 \| 1  13.42 [3.59-50.13]  4.82 [1.68-13.78] \| <0.001    0.003 \| - \| - \| - \| - \| - \| - \| \| **HIV-transmission risk group**  MSM  Heterosexual contacts  PWID  ICGE \| 1  2.59 [1.15-5.83]  0.54 [0.07-4.20]  1.37 [0.46-3.93] \| 0.021    0.553  0.563 \| 1  2.76 [0.91-8.52]  0    2.01 [0.49-8.22] \| 0.073    0.979  0.333 \| 1  1.72 [0.50-5.93]  1.26 [0.15-10.93]  0.73 [0.15-3.64] \| 0.393    0.831  0.696 \| - \| - \| - \| - \| - \| - \| \| **CD4+ cell count (cells/µl)^2^**  ≥200  <200 \| 1  3.22  [1.67-6.14] \| <0.001 \| 1  6.00  [2.44-14.74] \| <0.001 \| 1  1.57 [0.63-3.88] \| 0.331 \| 1  6.64   [1.74-25.28] \| 0.006 \| 1  3.38   [1.03-11.14] \| 0.045 \| 1  2.43  [0.88-6.74] \| 0.089 \| \| **Viral load (log10 copies/ml)^2^**  < 5  ≥ 5 \| 1  1.98 [1.05-3.75] \| 0.035 \| 1  1.72 [0.72-4.12] \| 0.227 \| 1  2.29 [0.91-5.73] \| 0.078 \| 1  1.97   [0.57-6.88] \| 0.286 \| 1  2.63  [0.89-7.76] \| 0.079 \| 1  1.49  [0.50-4.43] \| 0.470 \| \| **Antiretroviral therapy^1^**  Received  Not received \| 1  17.06   [9.52-30.55] \| <0.001 \| - \| - \| - \| - \| 1  17.89 [5.39-59.43] \| <0.001 \| 1  19.94 [7.15-55.58] \| <0.001 \| 1  15.65  [6.08-40.24] \| <0.001 \| \| ^1^, at any time before diagnosis of tuberculosis; ^2^, start of observation being defined as 01.01.2006 or HIV initial diagnosis date if later than 01.01.2006  ART, antiretroviral therapy; MSM, men who have sex with men; PWID, people who inject drugs; ICGE, immigrants from countries with generalised HIV epidemic  Cox regression, status=infection with tuberculosis, significance level 5%; HR, hazard ratio; [95%], 95% confidence interval \| \| \| \| \| \| \| \| \| \| \| \| \| |
| --- | --- | --- | --- | --- | --- | --- | --- | --- | --- | --- | --- | --- | --- | --- | --- | --- | --- | --- | --- | --- | --- | --- | --- | --- | --- | --- | --- | --- | --- | --- | --- | --- | --- | --- | --- | --- | --- | --- | --- | --- | --- | --- | --- | --- | --- | --- | --- | --- | --- | --- | --- | --- | --- | --- | --- | --- | --- | --- | --- | --- | --- | --- | --- | --- | --- | --- | --- | --- | --- | --- | --- | --- | --- | --- | --- | --- | --- | --- | --- | --- | --- | --- | --- | --- | --- | --- | --- | --- | --- | --- | --- | --- | --- | --- | --- | --- | --- | --- | --- | --- | --- | --- | --- | --- | --- | --- | --- | --- | --- | --- | --- | --- | --- | --- | --- | --- |

**B**

| \| **Predictor/risk variable for falling ill with tuberculosis** \| **Total patients** \| \| **Patients never started ART** \| \| **Patients on ART^1^** \| \| **TB incidence**  **<10/100,000** \| \| **TB incidence**  **10-50/100,000** \| \| **TB incidence**  **>50/100,000** \| \| \| --- \| --- \| --- \| --- \| --- \| --- \| --- \| --- \| --- \| --- \| --- \| --- \| --- \| \| HR [95%] \| *p-*  value \| HR [95%] \| *p-*  value \| HR [95%] \| *p-*  value \| HR  [95%] \| *p- value* \| HR  [95%] \| *p-*  *value* \| HR  [95%] \| *p-*  *value* \| \| **Sex**  Female  Male \| 1  1.00 [0.51-1.95] \| 0.999 \| 1  0.80 [0.33-1.97] \| 0.633 \| 1  1.30  [0.47-3.62] \| 0.610 \| 1  0.93 [0.22-4.06] \| 0.928 \| 1  6.71 [0.49-92.91] \| 0.155 \| 1  0.89 [36-2.18] \| 0.802 \| \| **TB incidence in country of origin**  **(per 100,000)**  <10  10-50      >50 \| 1  5.78  [2.55-13.12]  8.43  [4.05-17.54] \| <0.001  <0.001 \| 1  9.89  [2.99-32.77]  10.39 [3.40-31.77] \| <0.001  <0.001 \| 1  2.81  [0.76-10.40]  8.04 [2.76-23.45] \| 0.122  <0.001 \| - \| - \| - \| - \| - \| - \| \| **HIV-transmission risk group**  MSM  Heterosexual contacts  PWID  ICGE \| 1  2.58 [1.16-5.76]  0.48 [0.06-3.75]  1.36 [0.50-3.68] \| 0.021    0.484  0.551 \| 1  2.34 [0.78-6.99]  0    1.49 [0.41-5.42] \| 0.128    0.980  0.548 \| 1  2.34 [0.71-7.74]  1.43 [0.17-12.23]  1.13 [0.24-5.26] \| 0.165    0.746  0.874 \| - \| - \| - \| - \| - \| - \| \| **CD4+ cell count (cells/µl)^2^**  ≥200  <200 \| 1  2.80  [1.46-5.37] \| 0.002 \| 1  4.81  [1.96-11.80] \| 0.001 \| 1  1.58 [0.64-3.87] \| 0.319 \| 1  3.32 [1.00-11.01] \| 0.050 \| 1  8.40 [1.49-47.48] \| 0.016 \| 1  1.90 [0.76-4.77] \| 0.170 \| \| **Viral load (log10 copies/ml)^2^**  < 5  ≥ 5 \| 1  1.98 [1.03-3.82] \| 0.041 \| 1  1.73 [0.68-4.42] \| 0.252 \| 1  2.23 [0.90-5.53] \| 0.084 \| 1  1.54 [0.47-5.05] \| 0.479 \| 1  0.75 [0.13-4.42] \| 0.746 \| 1  3.23 [1.29-8.11] \| 0.013 \| \| **Antiretroviral therapy^1^**  Received  Not received \| 1  16.31   [9.15-29.07] \| <0.001 \| - \| - \| - \| - \| 1  8.02 [2.56-25.17] \| <0.001 \| 1  42.47 [9.38-192.20] \| <0.001 \| 1  18.27 [8.06-41.39] \| <0.001 \| \| ^1^, at any time before diagnosis of tuberculosis; ^2^, start of observation being defined as 01.01.2006 or HIV initial diagnosis date if later than 01.01.2006  ART, antiretroviral therapy; MSM, men who have sex with men; PWID, people who inject drugs; ICGE, immigrants from countries with generalised HIV epidemic \| \| \| \| \| \| \| \| \| \| \| \| \| |
| --- | --- | --- | --- | --- | --- | --- | --- | --- | --- | --- | --- | --- | --- | --- | --- | --- | --- | --- | --- | --- | --- | --- | --- | --- | --- | --- | --- | --- | --- | --- | --- | --- | --- | --- | --- | --- | --- | --- | --- | --- | --- | --- | --- | --- | --- | --- | --- | --- | --- | --- | --- | --- | --- | --- | --- | --- | --- | --- | --- | --- | --- | --- | --- | --- | --- | --- | --- | --- | --- | --- | --- | --- | --- | --- | --- | --- | --- | --- | --- | --- | --- | --- | --- | --- | --- | --- | --- | --- | --- | --- | --- | --- | --- | --- | --- | --- | --- | --- | --- | --- | --- | --- | --- | --- | --- | --- | --- | --- | --- | --- | --- | --- | --- | --- | --- | --- |

Cox regression, status=infection with tuberculosis, significance level 5%; HR, hazard ratio; [95%], 95% confidence interval

**Supplemental Table 2:** Hazard Ratio for people living with HIV/AIDS to develop tuberculosis in the Cologne/Bonn Cohort, 2006-2017, **A** using the covariates Germany, Sub-Saharan Africa and other countries for the origin, **B** using the TB incidences of the countries of origin as covariates in the Cox Regression of survival.

| **A) Total** |  | |  | | |  |
| --- | --- | --- | --- | --- | --- | --- |
| **Period** | **Sum patient-years** | | **TB cases** | | | **IDR [95%]** |
| 01.01.2006-31.12.2009 | 10,513.64 | | 28 | | | 0.266 [0.177-0.385] |
| 01.01.2010-31.12.2013 | 11,351.38 | | 17 | | | 0.150 [0.087-0.240] |
| 01.01.2014-31.12.2017 | 11,271.12 | | 15 | | | 0.133 [0.075-0.220] |
|  |  | |  | | |  |
| **B) HIV diagnosis before 01.01.2006** | | |  | | |  |
| **Period** | **Sum patient-years** | | **TB cases** | | | **IDR [95%]** |
| 01.01.2006-31.12.2009 | 8,235.99 | | 13 | | | 0.158 [0.084-0.270] |
| 01.01.2010-31.12.2013 | 6,517.54 | | 8 | | | 0.123 [0.053-0.242] |
| 01.01.2014-31.12.2017 | 4,543.67 | | 2 | | | 0.066 [0.014-0.193] |
|  |  | |  | | |  |
| **C) HIV diagnosis after 01.01.2006** | | |  | | |  |
| **Period** | **Sum patient-years** | | **TB cases** | | | **IDR [95%]** |
| 01.01.2006-31.12.2009 | 1,673.45 | | 15 | | | 0.896 [0.502-1.479] |
| 01.01.2010-31.12.2013 | 4,149.20 | | 9 | | | 0.217 [0.099-0.412] |
| 01.01.2014-31.12.2017 | 5,419.44 | | 13 | | | 0.240 [0.128-0.410] |
|  |  | |  | | |  |
| **D) Under ART** | | |  | | |  |
| **Period** | **Proportion of VL<50 copies/ml at enrollment** | **Sum**  **patient-years** | | **TB cases** | **IDR [95%]** | |
| 01.01.2006-31.12.2009 | 1,438/2,982 (48%) | 9,650.82 | | 15 | 0.155 [0.087-0.256] | |
| 01.01.2010-31.12.2013 | 1,305/3,161 (41%) | 10,590.11 | | 8 | 0.076 [0.033-0.149] | |
| 01.01.2014-31.12.2017 | 1,180/3,185 (37%) | 10,597.56 | | 2 | 0.019 [0.002-0.068] | |

ART, antiretroviral therapy; VL, viral load; IDR, Incidence density Rate (cases per 100 patient-years of observation); [95%], 95% confidence interval

**Supplemental Table 3:** Incidence Density Rate (IDR) stratified by period for the total cohort (A), the time of HIV diagnosis before (B) or after (C) 01.01.2006 and persons under antiretroviral therapy (ART) (D).


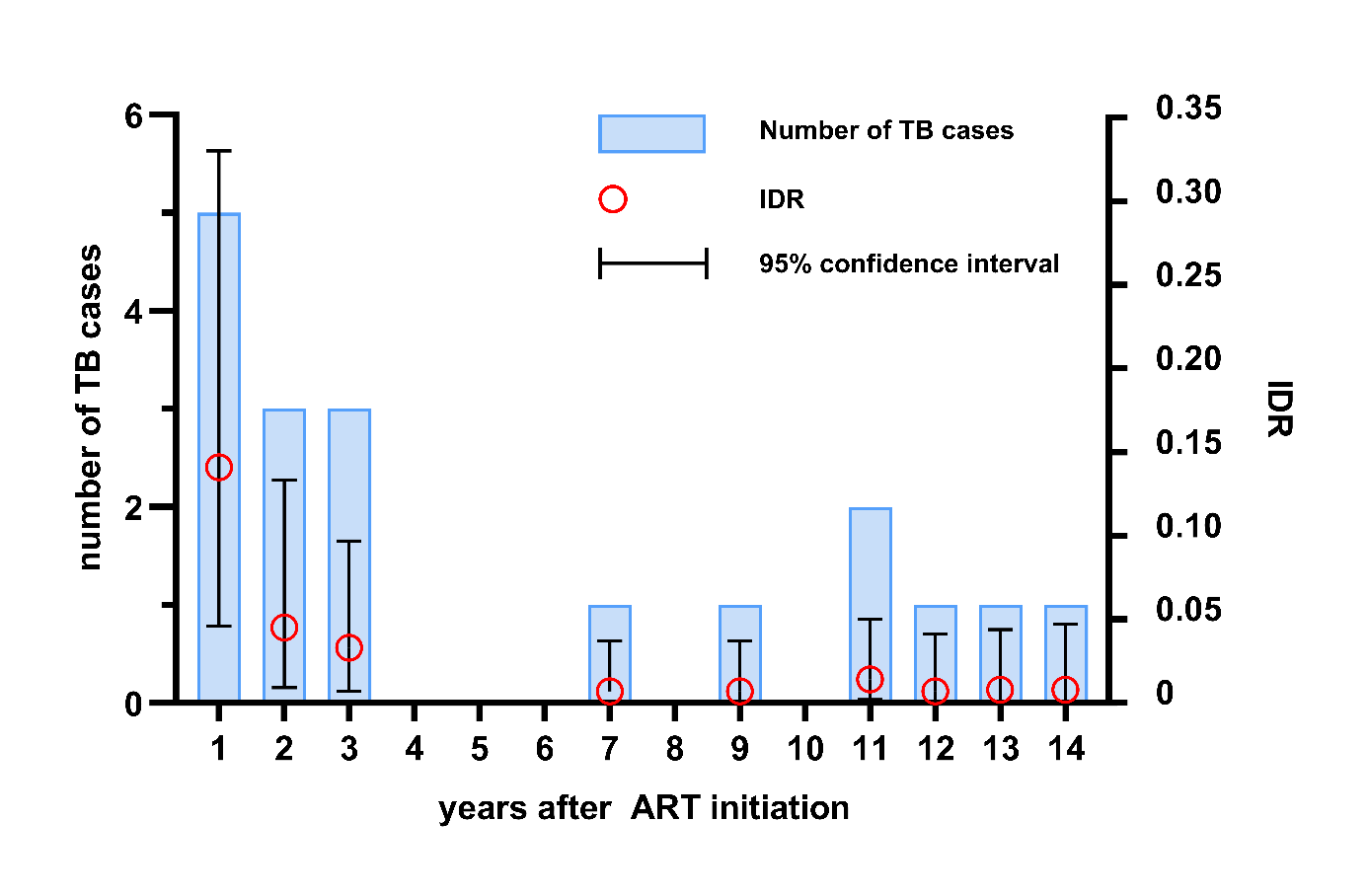


**Supplemental Figure 1:** Incidence Density Rate (IDR) stratified by time after initiation of antiretroviral therapy (ART).

HIV/TB co-infected persons whose TB diagnosis was made within 90 days of starting ART (n=35), persons with an observation period <90 days after ART initiation (n=90) and persons who started ART before 1996 (n=283) were excluded; missing data n=625.

IDR [cases per 100 patient-years of observation]
